# Inhibition of matrix stiffness relating integrin β1 signaling pathway inhibits tumor growth in vitro and in hepatocellular cancer xenografts

**DOI:** 10.1186/s12885-021-08982-3

**Published:** 2021-11-25

**Authors:** Changsong Wang, Xiaozhong Jiang, Bin Huang, Wenhao Zhou, Xiao Cui, Chenghong Zheng, Fenghao Liu, Jieling Bi, Yi Zhang, Hong Luo, Lin Yuan, Jianyong Yang, Yu Yu

**Affiliations:** 1 Department of Hepatopancreatobiliary Surgery, the Second People’ s Hospital of Yibin, Yibin, Sichuan 644000 P.R. China; 2 Center for Diagnosis and Treatment of Digestive Diseases, the Second People’ s Hospital of Yibin, Yibin, Sichuan 644000 P.R. China

**Keywords:** Matrix stiffness, Integrin β1, ERK1/2, Hepatocellular cancer

## Abstract

**Background:**

Cancer development is strictly correlated to composition and physical properties of the extracellular matrix. Particularly, a higher matrix stiffness has been demonstrated to promote tumor sustained growth. Our purpose was to explore the role of matrix stiffness in liver cancer development.

**Methods:**

The matrix stiffness of tumor tissues was determined by atomic force microscopy (AFM) analysis. In vitro, we used a tunable Polyacrylamide (PA) hydrogels culture system for liver cancer cells culture. The expression level of integrin β1, phosphorylated FAK, ERK1/2, and NF-κB in SMMC-7721 cells was measured by western blotting analysis. We performed MTT, colony formation and transwell assay to examine the tumorigenic and metastatic potential of SMMC-7721 cells cultured on the tunable PA hydrogels. SMMC-7721 cancer xenografts were established to explore the anticancer effects of integrin inhibitors.

**Results:**

Our study provided evidence that liver tumor tissues from metastatic patients possessed a higher matrix stiffness, when compared to the non-metastatic group. Liver cancer cells cultured on high stiffness PA hydrogels displayed enhanced tumorigenic potential and migrative properties. Mechanistically, activation of integrin β1/FAK/ ERK1/2/NF-κB signaling pathway was observed in SMMC-7721 cells cultured on high stiffness PA hydrogels. Inhibition of ERK1/2, FAK, and NF-κB signaling suppressed the pro-tumor effects induced by matrix stiffness. Combination of chemotherapy and integrin β1 inhibitor suppressed the tumor growth and prolonged survival time in hepatocellular cancer xenografts.

**Conclusion:**

A higher matrix stiffness equipped tumor cells with enhanced stemness and proliferative characteristics, which was dependent on the activation of integrin β1/FAK/ERK1/2/NF-κB signaling pathway. Blockade of integrin signals efficiently improved the outcome of chemotherapy, which described an innovative approach for liver cancer treatment.

**Supplementary Information:**

The online version contains supplementary material available at 10.1186/s12885-021-08982-3.

## Background

Hepatic carcinoma is one of the most common malignant carcinomas with a high mortality worldwide. The incidence of primary hepatocellular cancer is increasing in several centuries, however, limited clinical interventions are developed for patients with advanced liver cancer [1, 2]. Despite the advance of surgical excision and adjuvant chemotherapy, a number of liver cancer patients suffer the sustained tumor growth and tissue infiltration during treatment [3]. Currently, the underlying mechanism of liver cancer development remains poorly understood. Therefore, there is an urgent demand to elucidate the underlying mechanism of tumor occurrence and develop innovative strategy to impair tumor growth.

Cancer development is bound up with diverse biological factors, including immunosuppression, inflammation reaction, pro-survival cytokines secreted by stromal cells, tumor heterogeneity and extracellular matrix [4–6]. Compelling findings provide evidence that the extracellular matrix could support tumor cells proliferation and epithelial-mesenchymal transition, thereby causing the tumor growth and distant metastasis [7]. The extracellular matrix in tumor tissues typically consists of abundant levels of collagen and fibrinogen, which could be further crosslinked by soluble mediators, such as lysyl oxidase, eventually generating matrix stiffness [8]. As previously reported, matrix stiffness induced mechanical force has been emerged as critical determinant of cancer progression in many solid tumor types [9]. And matrix stiffness is increasingly appreciated as an important mediator of cancer metastasis and development [10]. Increasing evidence implicates that greater matrix stiffness could have profound effects on cancer progression, including oncogenic intracellular signals activation and upregulation of pro-survival signaling [11]. Several pro-survival signaling pathways, including FAK/AKT and β-catenin signaling pathways, have been demonstrated to be involved in the matrix stiffness associated tumor development [12, 13]. Matrix stiffness not only impacts cancer cell proliferation, but its effects similarly extend to promote cell migration and invasion to surrounding tissues. AJ Rice and his colleagues reported that greater matrix stiffness could mediate the epithelial-mesenchymal transition process of pancreatic cancer cells, resulting in the tumor cells invasion and distant metastasis [14]. Those studies support matrix stiffness as a therapeutic target for cancer treatment. Intriguingly, mechanical force induced by matrix stiffness has long been a focus of hepatology, and portal pressure is emerged as the target in end-stage liver disease therapy [15, 16]. However, the underlying mechanism of matrix stiffness promoting cancer development remains controversial, and it might be a feasible strategy to target matrix stiffness associated signaling pathway for clinical liver cancer therapy.

Owing to the essential role of matrix stiffness in cancer development, we examined the matrix stiffness in low/high degree malignant liver cancer tissues and further explored the underlying mechanism. Our study confirmed that greater matrix stiffness could promote the stem-like phenotypes in liver cancer cells, thereby resulting in the sustained tumor growth and cell invasion. Additionally, we further determined the underlying mechanism of matrix stiffness associated tumor progression, which was dependent on an integrin β1/FAK/ERK1/2/NF-κB signaling pathway. Blockade of integrin signals efficiently suppressed the activation of pro-survival signaling pathway induced by matrix stiffness, leading an improved anticancer effect, and providing novel target for clinical liver cancer treatment.

## Methods

### Cell lines culture and regents

The human hepatocarcinoma cell (HCC) lines SMMC-7721 and HepG2 were purchased from the American Type Culture Collection (ATCC, Rockville, MD). All cell lines were cultured in Roswell Park Memorial Institute (RPMI) 1640 complete medium supplemented with 10% fetal bovine serum (Gibco, MA, USA) at 37 °C in a 95% humidified atmosphere containing 5% CO_2_. The inhibitors, such as SCH772984, PF-573228, JSH-23 were purchased from Selleck Chemicals (MA, USA). GLPG0187 was purchased from MCM (MA, USA). The chemotherapeutic agents Adriamycin (ADM) and cis-diamine dichloro platinum (DDP) were purchased from Sangon (Shanghai, China).

### Cell proliferation

The cell proliferation was examined by MTT assay. Briefly, a density of 2 × 10^3^ per/well SMMC-7721 and HepG2 tumor cells were seeded in 96-well plates and cultured with RPMI 1640 complete culture medium supplemented with 10% fetal bovine serum. Cell proliferation was examined at 0, 24, 48, and 72 h. Samples were added with 10 μl MTT (5 mg/ml) and incubated for 4 h at 37 °C. The formazan crystals in the cells were solubilized with stop solution (100 μl/well). Subsequently, the samples were analyzed at 570 nm using a Microplate Reader Model 550 (BIO-RAD, Shanghai, China).

### Colony formation

SMMC-7721 and HepG2 cells were seeded in 6-well plates at 200 cells per well and cultured in RMPI complete medium in a humidified incubator. After 14 days, colonies were fixed with paraformaldehyde and stained with crystal violet (Beyotime, Beijing, China). Visible colonies were counted. Each experiment was performed three times independently.

### Cell invasion ability detection by transwell assay

The cell suspension (1 × 10^6^ cells /ml, 200 μl) was added into the upper insert of a transwell chamber (8 μm, Corning, USA), and RMPI 1640 complete culture medium containing 15% fetal calf serum was added to the lower chamber. After 24 h, the migrating cells were fixed with 4% paraformaldehyde and stained with 0.1% crystal violet. Then the migrating cells numbers were counted. Each experiment was performed three times independently.

### Preparation of different stiffness PA gel

PA gels used in cell culture with different stiffness were prepared as indicated in previous studies [17]. Briefly, the coverslips were coated with a thin layer of gel containing a mixture of 3 to 10% acrylamide and 0.01-0.3% bis-acrylamide, producing gels of 2, 8 and 20 kPa stiffness. Addition of 10% APS (1/100 volume) and TEMED (3/1000 volume) promoted the PA gel polymerization. Then the coverslips were washed with PBS twice for 20 min, followed by sterilizing in PBS solution for 1 h with ultraviolet light. Next, 50 μl heterobifunctional sulphosuccinimidyl 6-(4′-azido-2′-nitrophe-nylamino) hexanoate were added and photo-activated for 5 min with ultraviolet light. Then, the coverslips were coated with 10 μl/ml fibronectin for 1.5 h and rinsed before cell seeding. Gels were soaked in serum-free culture media for 24 h for usage. All regents of PA gels were purchased from Solarbio (Beijing, China).

### Patients’ tumor tissues samples

Formalin-fixed, paraffin-embedded human hepatocarcinoma tumor tissue sections were obtained from the Second People’ s Hospital of Yibin (Sichuan, China), and were divided into low degree malignant group (LD, stage A ~ B) and high degree malignant group (HD, stage C ~ D) according to the Barcelona Clinic Liver Cancer criterion. The protocols were approved by Regional Scientific Ethics Committee of the Second People’ s Hospital of Yibin (Sichuan, China). Written informed consent was attained from all subjects, and all methods were performed in accordance with the Declaration of Helsinki.

### AFM analysis

Atomic force microscopy and analysis were performed as previously indicated [18]. Frozen tissue blocks were cut into 20 μm thick sections. All the sample sections were immersed in PBS at room temperature before AFM measurement. The samples were maintained in proteinase inhibitor in PBS (protease inhibitor cocktail, Roche 14 Diagnostics, 11,836,170,001) and supplemented with Propidium Iodide (SIGMA P4170, 20 μg/ml) during the AFM session. MFP3D-BIO inverted optical AFM (Asylum Research) mounted on a Nikon TE2000-U inverted fluorescent microscope was used for AFM analyze as previously described. Ten frozen sections were analyzed in each tumor tissue, and 10 tumor tissues were collected and examined in each group.

### Western blot analysis

The total proteins were extracted by radioimmunoprecipitation assay (RIPA) lysis buffer (Beyotime, Shanghai, China) with the protease inhibitor phenylmethylsulfonyl fluoride (PMSF) (Beyotime, Shanghai, China). Cell lysates were separated on 10% sodium dodecyl sulfate-polyacrylamide gels (SDS-PAGE), then blocked and incubated with the corresponding primary antibodies: anti-integrin β1 (1:1000, Abcam, Cambridge, UK), anti-phosphorylated FAK (1:1000, Abcam, Cambridge, UK), anti-FAK (1:1000, Abcam, Cambridge, UK), anti- phosphorylated ERK1/2 (1:1000, Abcam, Cambridge, UK), anti-ERK1/2 (1:1000, Abcam, Cambridge, UK), anti-NF-κB (1:1000, Abcam, Cambridge, UK). Subsequently, samples were incubated with an HRP-conjugated secondary antibody (1:1000, Abcam, Cambridge, UK). β-actin served as an internal control.

### Immunofluorescence staining

The sections of tumor tissues were dewaxed, rehydrated, quenched of endogenous peroxidase, blocked by 5% BSA, and incubated with the primary antibodies: anti-p-FAK, anti-p-ERK, anti-NF-κB (1:200, Abcam, Cambridge, UK) overnight at 4 °C, and followed by signal amplification using the ABC HRP Kit (Thermo, MA, USA) for 2 h at room temperature, and the nucleus was stained with DAPI. All immunofluorescence images were captured by FV1000 confocal microscope (Leica, Barnack, Germany) and the intensity of protein expression was calculated by image J software.

### Real-time PCR

Total RNA was extracted from tumor cells using TRIzol (Thermo, MA, USA) according to the manufacturer’s protocol. The quantification of mRNA levels was conducted by real-time PCR using SYBR green dye (Thermo, MA, USA). And 1μg cDNA was used as template for amplification. GAPDH was used as the internal control and normalized the target gene level to the GAPDH by the ΔΔC_t_ method to quantify the relative expression. The primer pairs in our study were used as follow: integrin β1: Forward, 5′- CCTACTTCTGCACGATGTGATG-3′, and reverse, 5′- CCTTTGCTACGGTTGGTTACATT-3′; integrin β2: Forward, 5′- TGCGTCCTCTCTCAGGAGTG-3′, and reverse, 5′-GGTCCATGATGTCGTCAGCC-3′; integrin β3: Forward, 5′-GTGACCTGAAGGAGAATCTGC-3′, and reverse, 5′-CCGGAGTGCAATCCTCTGG-3′; integrin β4: Forward, 5′-GCAGCTTCCAAATCACAGAGG-3′, and reverse, 5′-CCAGATCATCGGACATGGAGTT-3′; integrin β5: Forward, 5′-TCTCGGTGTGATCTGAGGG-3′, and reverse, 5′-TGGCGAACCTGTAGCTGGA-3′; integrin β6: Forward, 5′-TCCATCTGGAGTTGGCGAAAG-3′, and reverse, 5′-TCTGTCTGCCTACACTGAGAG-3′; integrin β7: Forward, 5′-AGAATGGCGGAATCCTCACCT-3′, and reverse, 5′-TGAAGTTCAGTTGCTTGCACC-3′; integrin β8: Forward, 5′-ACCAGGAGAAGTGTCTATCCAG-3′, and reverse, 5′-CCAAGACGAAAGTCACGGGA-3′. Each experiment was performed three times independently.

### Flow cytometry

The SMMC-7721 and HepG2 tumor cells were collected and fixed with 4% paraformaldehyde, washed by PBS for three times. Subsequently, the cells were stained with anti-human CD133 (Biolegend, MA, USA) at a dilution of 1:500 at 4 °C. Human IgG Isotype was stained as control at a dilution of 1:500 in flow cytometry analysis. After 30 min, samples were washed by PBS and were examined using an AccuriC6 (BD, MA, USA). FlowJo software 2.0 (Biolegend, MA, USA) was used for analysis. Each experiment was performed in three independent times.

### Si RNA interference

For integrin β1 or NF-κB silence, SMMC-7721 and HepG2 cells were infected with Lipofectamine 8000 (Beyotime, Beijing, China) according to the manufacturer’s protocol. The relevant NF-κB siRNA sequence as followed: siRNA#1: 5′-GAAGGGTTGCCAACCAAGT-3′ and siRNA#2: 5′-GGTGGCTTTGATGCAATCA-3′;

### Animal experiments

Four to six weeks female nude mice were purchased from Huafukang company (Beijing, China), and raised in SPF level. The animal protocols were approved by the Animal Care and Use Committee of the Second People’ s Hospital of Yibin Committee (#2018-02-13), according to the National Institutes of Health Guide for the Care and Use of Laboratory Animals. The animal studies were conducted in accordance with the Public Health Service Policy and complied with the ARRIVE guidelines for the humane use and care of animals. For establishing subcutaneous hepatocarcinoma model, 2 × 10^6^ SMMC-7721 tumor cells in 100 ul PBS were subcutaneously inoculated on their armpits of right anterior limbs. All the mice were randomly divided into 4 group (6 mice per group). After 2 weeks, tumor bearing mice were treated with ADM (4 mg/kg), DDP (0.5 mg/kg), or DDP (0.5 mg/kg) combining GLPG0187 (i.p 100 mg/kg; dissolved in 2% DMSO in PBS), and 2% DMSO in PBS for control. Mice were treated every 3 days and the treatment lasted for 2 weeks. Tumor volume was measured using the formula: volume = length × width^2^/2. The survival time of mice was recorded. For tumorigenesis analysis, SMMC-7721 cells (2 × 10^5^) were subcutaneously injected into the right side of mice, and tumor numbers on each mouse was measured after 2 weeks. Mice were sacrificed by cervical dislocation.

### Statistical analysis

Results are presented as the mean ± standard deviation (SD), and the data in bar graphs are represented as the mean fold change relative to the untreated or control groups with SD of three independent experiments. Statistical significance between groups was calculated by Student’s t test for two groups or by one-way ANOVA for more than two groups using Graphpad 6.0. The log-rank (Mantel–Cox) test was used to analyze the long-term survival curve. The ARRIVE reporting guidelines were used to complete the analysis [19]. Statistical significance was set at *P* < 0.05. All error bars are expressed as the mean ± SD of three independent experiments.

## Results

### High matrix stiffness promotes liver cancer cells proliferation and invasion

**Fig. 1:**
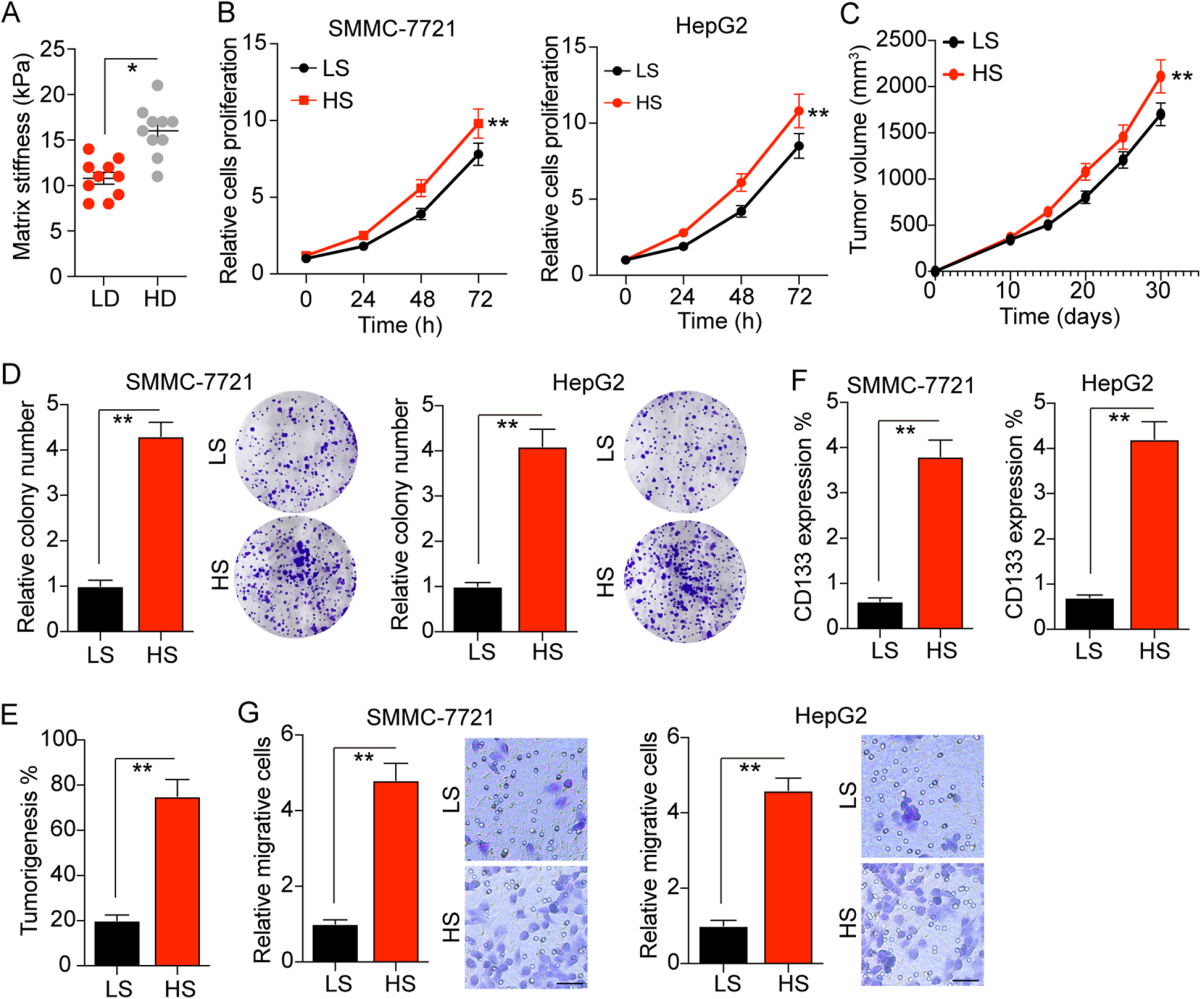
A higher matrix stiffness promotes liver cancer cells proliferation and invasion. **A** The matrix stiffness of tumor tissues from liver cancer patients with low degree malignant (LD, stage A ~ B) and high degree malignant (HD, stage C ~ D), examined by AFM analysis. **B** The relative cells proliferation of SMMC-7721 and HepG2 cells cultured on the tunable PA hydrogels with a stiffness of 12 kPa (LS, low stiffness) or 16 kPa (HS, high stiffness). **C** The tumor growth of SMMC-7721 cells, pre-cultured in tunable PA hydrogels with a stiffness of 12 kPa (LS) or 16 kPa (HS), in nude mice established as indicated, tumor volume was measured at the indicated time points (*n* = 6 mice per group). **D** The relative colony number of SMMC-7721 and HepG2 pre-cultured in tunable PA hydrogels with a stiffness of 12 kPa (LS) or 16 kPa (HS). **E** The percent of tumorigenesis of SMMC-7721 cells, pre-cultured in tunable PA hydrogels with a stiffness of 12 kPa (LS) or 16 kPa (HS), in nude mice established as indicated. **F** The CD133 expression of SMMC-7721 and HepG2 cells pre-cultured in tunable PA hydrogels with a stiffness of 12 kPa (LS) or 16 kPa (HS), measured by flow cytometry as indicated. **G** The relative migrative cells of SMMC-7721 and HepG2 cells pre-cultured in tunable PA hydrogels with a stiffness of 12 kPa (LS) or 16 kPa (HS). The scale bar is 30 μm. Data represent mean ± SD, *P < 0.05, **P < 0.01 or as indicated

Increasing evidence has suggested that matrix stiffness is tightly correlated to the cancer stem cells proliferation and tumor development in several tumor types [20–23]. Here, to explore the potential role of matrix stiffness in liver cancer, we isolated tumor tissues from clinical liver cancer patients, which were divided into low degree malignant group (LD, stage A ~ B) and high degree malignant group (HD, stage C ~ D) according to the Barcelona Clinic Liver Cancer criterion. Intriguingly, the tissues stiffness determined by AFM analysis suggested that those high degree malignant liver tumor tissues possessed a higher matrix stiffness (14 ~ 18 kPa), when compared to the LD group (8 ~ 15 kPa) (Fig. 1A). To further investigate the potential correlation between matrix stiffness and tumor progression in liver cancer, we seeded liver cancer cells, HepG2 and SMMC-7721, on the tunable PA hydrogels with a stiffness of 12 kPa (LS, low stiffness) or 16 kPa (HS, high stiffness). After culture of 7 days, we collected those liver cancer cells and seeded them into 96 plates for proliferation analysis. Of note, high matrix stiffness culture significantly strengthened the cells proliferation of SMMC-7721 and HepG2 (Fig. 1B). Consistently, high stiffness culture promoted the tumor growth of SMMC-7721 bearing nude mice in vivo (Fig. 1C). Our colony formation analysis revealed that SMMC-7721 and HepG2 cultured in high stiffness hydrogels exhibited a strengthened ability to form spheroid colonies (Fig. 1D). The same result was observed in tumorigenic analysis (Fig. 1E), suggesting that high matrix stiffness could promote the capability of proliferation and tumorigenic potential in liver cancer cells. It has been clarified that cancer stem cells are the major culprit to drive tumor growth and promote cancer relapse. Therefore, we isolated the PA hydrogels cultured liver cancer cells, SMMC-7721 and HepG2, to examine the liver cancer stem cells marker CD133. As anticipated, increasing expression of CD133 in high stiffness hydrogels cultured tumor cells was observed (Fig. 1F), indicating that high matrix stiffness promotes liver cancer stem cells. Accumulating studies illustrated that cancer stem cells are strictly correlated to the tumor invasion and distant metastasis in patients. Consistently, our transwell analysis indicated that SMMC-7721 and HepG2 cultured in high stiffness PA hydrogels exhibited enhanced capability of cells invasion (Fig. 1G). Together, these data provided evidence to suggest that high matrix stiffness could facilitate the proliferative properties and tumorigenic potential of tumor cells, resulting in a poor prognosis in liver cancer.

### Matrix stiffness mediates cancer cells proliferation through integrin β1

**Fig. 2:**
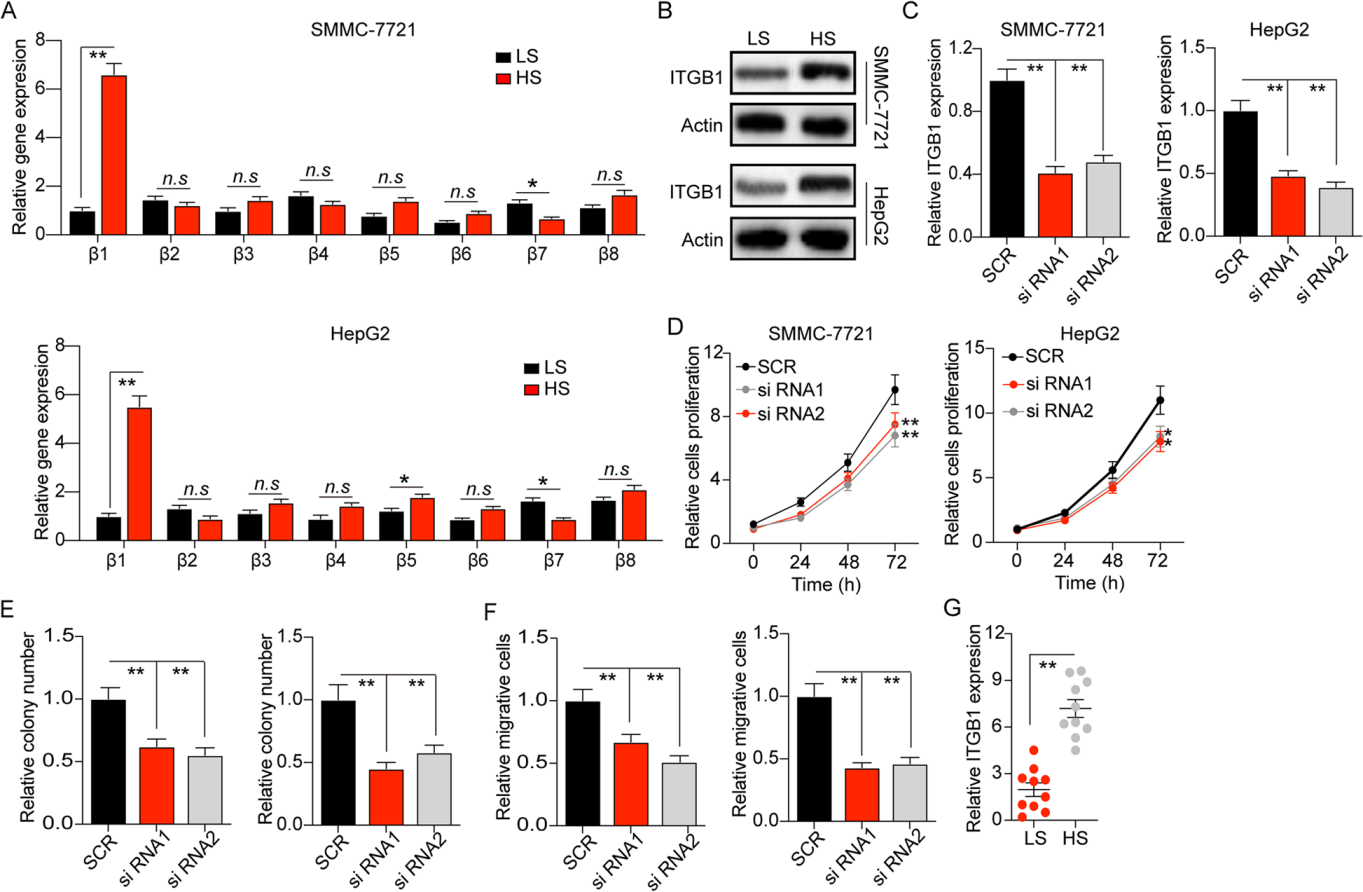
Matrix stiffness mediates cancer cells proliferation through integrin β1. **A** The relative gene expression of integrin β1, β2, β3, β4, β5, β6, β7, β8 in SMMC-7721 and HepG2 cells cultured in tunable PA hydrogels with a stiffness of 12 kPa (LS) or 16 kPa (HS), measured by q-PCR. **B** The protein expression level of integrin β1 in SMMC-7721 and HpeG2 cells cultured in matrix stiffness with 12 kPa (LS) or 16 kPa (HS), measured by western blot. **C** The relative integrin β1 expression level in SMMC-7721 and HepG2 cells with integrin β1silenced by siRNA or not, measured by q-PCR. **D** The relative cells proliferation of SMMC-7721 and HepG2 cells with integrin β1 silenced by siRNA1, siRNA2 or not, cultured in in tunable PA hydrogels with a stiffness of 16 kPa. **E** The relative colony number of SMMC-7721 and HepG2 cells pre-cultured in in tunable PA hydrogels with a stiffness of 16 kPa, with integrin β1 silenced by siRNA1, siRNA2 or not. **F** The relative migrative cells number of SMMC-7721 and HepG2 cells pre-cultured in in tunable PA hydrogels with a stiffness of 16 kPa, with integrin β1 silenced by siRNA1, siRNA2 or not. **G** The relative integrin β1 expression of tumor tissues with high matrix stiffness (HS) or low matrix stiffness (LS), from liver cancer patients, measured by real-time PCR. Data represent mean ± SD, *P < 0.05, **P < 0.01 or as indicated

Integrins are glycoproteins on cell surfaces, which are tightly involved in the adhesion of cells to cells or extracellular materials [19]. Accumulating studies have demonstrated that the expression of integrins in tumor cells are essential to the biodynamic signal transduction between extracellular materials and tumor cells [24]. Here, we examined the expression of integrin β family (integrin β1 ~ 8) in our PA hydrogels cultured tumor cells. The expression of *ITGB1* was obviously up-regulated in high stiffness PA hydrogels cultured SMMC-7721 and HepG2 cells (Fig. 2A). The elevated expression of integrin β1 was observed in protein level (Fig. 2B). To further explore the role of integrin β1 in liver cancer development, we used siRNA to silence integrin β1 in SMMC-7721 and HepG2 cells (Fig. 2C). Those integrin β1 silenced SMMC-7721 and HepG2 cells exhibited weakened capability of cells proliferation (Fig. 2D) and colony formation (Fig. 2E), despite high matrix stiffness culture. Meanwhile, cell invasion induced by matrix stiffness was inhibited when integrin β1 silence (Fig. 2F). Intriguingly, silence of ITGB1 in SMMC-7721 cells (low stiffness culture) showed limited effects on the cell proliferation, colony formation or cell migration (Fig. S1A, B and C), suggesting that high matrix promoted liver cancer development through an integrin β1 dependent manner. We further examined the *ITGB1* expression (mRNA level) in tumor tissues from patients, and found increasing expression of *ITGB1* in tumor tissues from liver cancer patients with a higher tumor matrix stiffness (Fig. 2G). Those results implicated that matrix stiffness promotes liver cancer development through integrin β1.

### Matrix stiffness facilitates activation of FAK/ERK/NF-κB signaling pathway through integrin β1

**Fig. 3:**
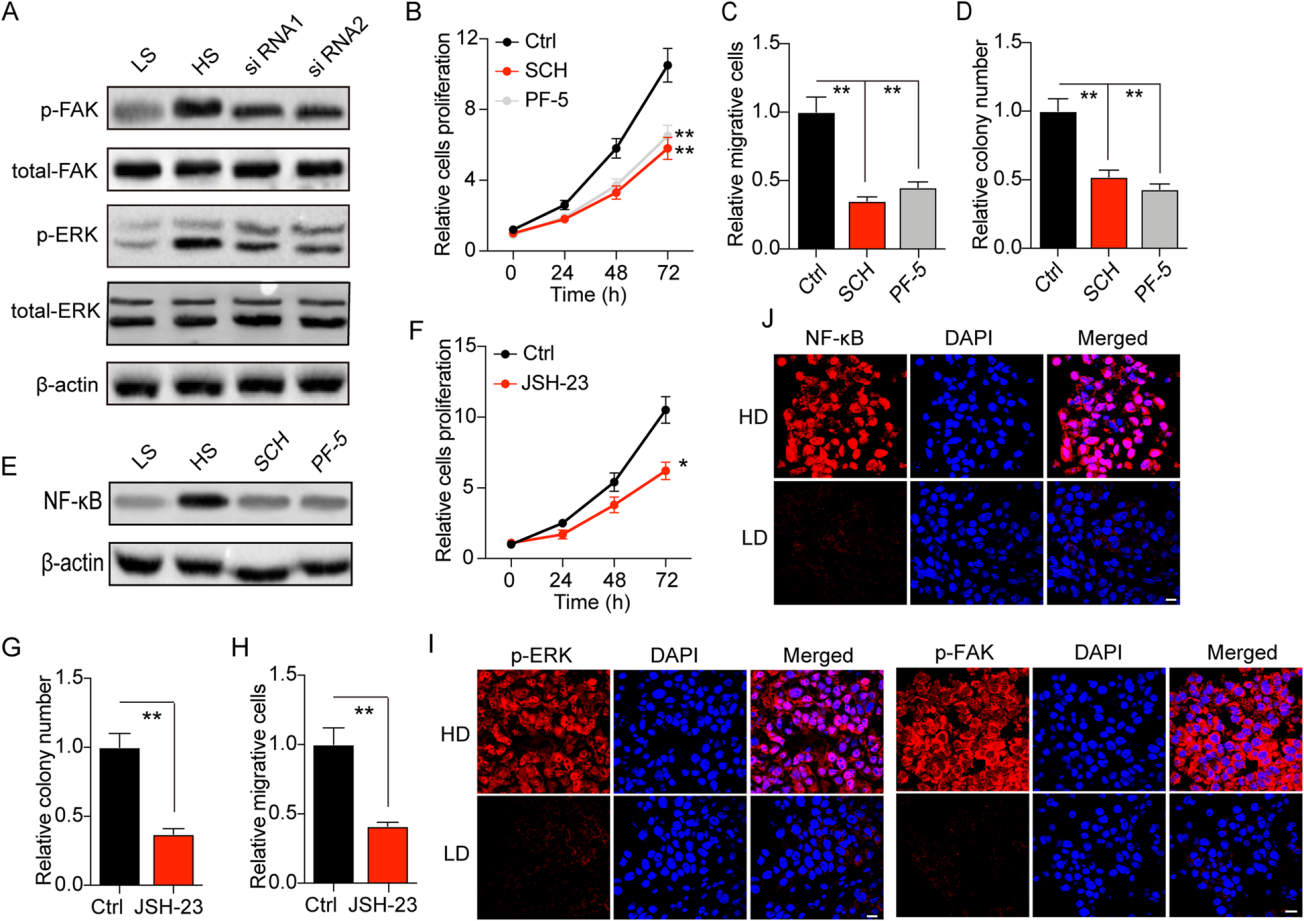
Matrix stiffness facilitates activation of FAK/ERK/NF-κB signaling pathway through integrin β1. **A** The protein level of p-FAK, total FAK, p-ERK1/2, total ERK in SMMC-7721 cells cultured in tunable PA hydrogels with low stiffness (12 kPa) and high stiffness (16 kPa), as well as the integrin β1 silenced SMMC-7721 cells cultured in high stiffness, measured by western blotting. **B**-**D** The relative cells proliferation (**B**), the relative migrative cells number (**C**), the relative colony number (**D**) of SMMC-7721 cells treated with PBS, SCH772984 (2 nM) or PF-573228 (3 nM) respectively, cultured in high matrix stiffness. **E** The protein expression level of NF-κB in SMMC-7721 cells cultured in tunable PA hydrogels with low stiffness (12 kPa) and high stiffness (16 kPa), as well as the integrin β1 silenced SMMC-7721 cells cultured in high stiffness, measured by western blot. **F**-**H** The relative cells proliferation (**F**), the relative migrative cells number (**G**), the relative colony number (**H**) of SMMC-7721 cells treated with PBS, JSH-23 (5 μM) respectively, cultured in high matrix stiffness. **I**, **J** Immunofluorescence analysis of phosphorylated FAK, ERK1/2 (**I**) and NF-κB (**J**) in tumor tissues from tumor tissues with high degree malignant (HD) or low degree malignant; scale bar, 20 μm. Data represent mean ± SD, *P < 0.05, **P < 0.01 or as indicated

Integrins are transmembrane receptors that response to the RGD modification on its ligands, leading to activation of diverse pro-survival signaling pathways, including the PI3K/AKT signaling pathway, JAK/STAT3 and FAK/ERK signaling pathway [24, 25] Consistently, we observed elevated expression of phosphorylated FAK and ERK1/2 in high stiffness PA hydrogels cultured SMMC-7721 and HepG2 cells (Fig. 3A and S1A). Silence of integrin β1 by siRNA suppressed FAK/ERK activation in high stiffness PA hydrogels cultured SMMC-7721 and HepG2 cells (Fig. 3A and S2A), indicating that matrix stiffness mediated the activation of FAK/ERK signaling pathway through integrin β1 in liver cancer cells. To further determine the role of FAK/ERK signal in liver cancer development, we used ERK1/2 inhibitor SCH772984 and FAK inhibitor PF-573228 to treat high stiffness PA hydrogels cultured tumor cells [26, 27]. Intriguingly, suppression of FAK/ERK signals efficiently retarded the proliferation (Fig. 3B and S2B), colony formation (Fig. 3C and S2C) and invasion (Fig. 3D and S2D) of SMMC-7721 and HepG2 cells cultured by high stiffness PA hydrogels. However, limited tumor suppressive effects of SCH772984 and PF-573228 were observed in low stiffness cultured SMMC-7721 cells (Fig. S2E, F and G). Those results suggested that high matrix stiffness contributed to the activation of FAK/ERK signals, leading to the cell proliferation and invasion in liver cancer. Compelling finding has demonstrated that NF-κB serves as the downstream molecule of FAK, which is tightly involved in tumor growth and invasion [28, 29]. Notably, we observed elevated expression of NF-κB expression in high stiffness PA hydrogels cultured SMMC-7721 and HepG2 cells, whereas blockade of FAK/ERK signaling obviously suppressed the expression of NF-κB (Fig. 3E and S2H), suggesting that high matrix stiffness up-regulated NF-κB through FAK/ERK signaling. Subsequently, we further used JSH-23, an NF-κB inhibitor, to treat high stiffness PA hydrogels cultured SMMC-7721 and HepG2 cells [30]. Blockade of NF-κB signaling suppressed the cells proliferation (Fig. 3F and S2I), colony formation (Fig. 3G and S2J) and invasion (Fig. 3H and S2K) of high stiffness PA hydrogels cultured tumor cells. However, limited tumor suppressive effects of JSH-23 were found in SMMC-7721 cultured in low stiffness gels (Fig. S2L, M and N). In consistent, the immunofluorescence staining implicated that elevated expression of phosphorylated FAK, ERK1/2 and NF-κB was found in high degree malignant tumor tissues, when compared to the low degree group (Fig. 3I and J). Taken together, those results suggested that high matrix stiffness could facilitate liver cancer progression through an integrin β1/FAK/ERK/ NF-κB signaling pathway.

### Blockade of integrin signals improved the outcome of chemotherapy in hepatocellular cancer xenografts

**Fig. 4:**
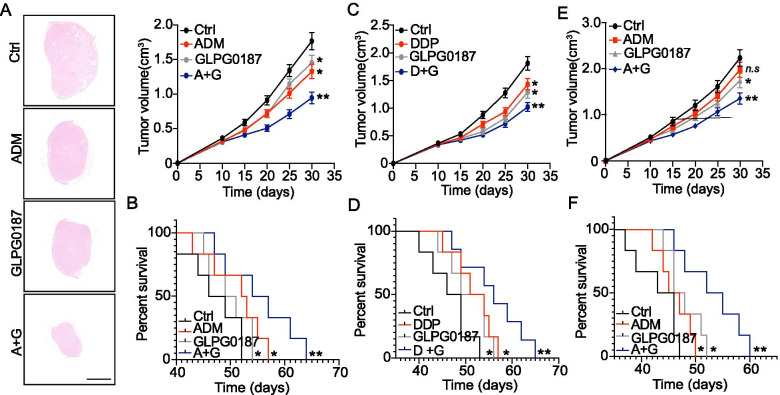
Blockade of integrin signals strengthened tumor suppressive effects of chemotherapy. **A**, **B** The tumor growth (**A**) and survival time (**B**) of SMMC-7721 cells in nude mice, established as indicated, treated with PBS, ADM, GLPG0187, ADM+ GLPG0187 respectively as indicated. (n = 6 per group). The scale bar in H&E staining is 2000 μm. **C**, **D** The tumor growth (**C**) and survival time (**D**) of SMMC-7721 cells in nude mice, established as indicated, treated with PBS, DDP, GLPG0187, DDP + GLPG0187 respectively as indicated. (n = 6 per group). **E**, **F** The tumor growth of SMMC-7721 cells, pre-cultured in tunable PA hydrogels with high stiffness (16 kPa), in nude mice established as indicated, then treated with PBS, ADM, GLPG0187, ADM+ GLPG0187 respectively as indicated. (n = 6 per group). Data represent mean ± SD, *P < 0.05, **P < 0.01 or as indicated

Owning to the essential role of integrin signaling in liver cancer, it might be feasible to suppress the integrin signals for improved anticancer effects. Here, we combined integrin inhibitor GLPG0187 with chemotherapeutic agents ADM/DDP for hepatocellular cancer xenografts. Notably, combination of GLPG0187 and ADM efficiently suppressed the tumor growth (Fig. 4A) and prolonged survival time (Fig. 4B) of SMMC-7721 bearing mice. The similar results were observed in DDP combination group (Fig. 4C and D). Those results suggested that suppression of integrin signals efficiently improved the outcome of chemotherapy in liver cancer. To further explore the anticancer effects of integrin inhibitor to high matrix stiffness cultured tumor cells, we seeded SMMC-7721 cells on 16 kPa PA hydrogels for 7 days. Those high stiffness cultured SMMC-7721 cells were subcutaneously injected into immunodeficient mice, and treated with ADM and GLPG0187. Intriguingly, limited tumor suppressive effects were observed in ADM treated group, which might be due to the stem-like phenotypes and potential drugs resistance caused by high stiffness culture. However, GLPG0187 or combination treatment obviously suppressed tumor growth, as well as prolonging the survival time of tumor bearing mice (Fig. 4E and F). Together, those results implicated that inhibiting integrin signals by GLPG0187 could efficiently improve the outcome of chemotherapy, which provided novel sight for adjuvant therapy in liver cancer treatment.

## Discussion

In this study, we sought to explore the potential correlation between matrix stiffness and liver cancer development. Using a tunable PA hydrogels culture system, we observed that liver cancer cells cultured in hydrogels with a higher matrix stiffness are prone to reveal strengthened stem like phenotypes and proliferative characteristics. Additionally, we confirmed the underlying mechanism of cancer development induced by high matrix stiffness culture, which was dependent on the activation of integrin β1/FAK/ERK1/2/NF-κB signaling pathway in tumor cells. Blockade of integrin signals could obviously enhance the tumor suppressive effects of chemotherapy in SMMC-7721 bearing mice, which presented a novel strategy for clinical liver cancer intervention.

As described in our study, extracellular matrix stiffness was able to promote the stem-like phenotypes and proliferative properties of liver cancer cell lines. And the capability of tumor cell to grow steadily in high matrix stiffness hydrogels might predict the tumor development in vivo. Accordingly, our AFM analysis implicated that liver cancer patients in high malignant group possessed greater matrix stiffness in tumor tissues. Those results suggested the essential role of matrix stiffness in regulating liver cancer development. Previous reports have demonstrated that matrix stiffness is involved in the tumor development in several cancer types, including breast cancer and glioma [31–33]. Apart from enhanced tumorigenic potential and cell proliferation induced by matrix stiffness, current studies provided evidence that matrix stiffness also facilitates epithelial-mesenchymal transition of lung cancer cells and promotes cancer metastasis [34–36]. Notably, cancer cells, such as pancreatic cells, that exhibited mesenchymal-like phenotypes, are prone to be resistant to chemotherapy [37, 38]. This explains our results in hepatocellular cancer xenografts, in which high matrix stiffness cultured SMMC-7721 revealed resistance to chemotherapy and suppression of matrix stiffness associated integrin signals reversed chemo-resistance. However, the potential role of matrix stiffness in determining cancer cells responses to therapeutic agents and the underlying mechanism of chemo-resistance in liver cancer remain to be developed.

Integrins are transmembrane receptors that are tightly involved in cell-extracellular matrix adhesion and extracellular chemical/biomechanical signal transduction [39]. Integrins on cellular surface could interact with the matrix elements, such as collagen and fibrin, to promote the activation of anti-apoptosis and pro-survival signaling pathways in tumor cells [40]. Increasing evidence suggested that diverse pro-survival signaling pathways are involved in the integrins associated tumor development, including PI3K/AKT, HAK/STAT and Wnt signaling pathway [24, 41, 42]. More importantly, matrix stiffness could mediate the activation of integrin signaling pathways, thereby resulting in enhanced tumor growth and cancer metastasis [12]. Consistent to previous findings, our study further confirmed the role of matrix stiffness in liver cancer, in which a greater matrix stiffness promoted liver cancer development through FAK/ERK1/2 signals activation and NF-κB nucleus translocation. In our study, we used FAK, ERK and NF-κB inhibitors to treat liver cancer cells, in which blockade of FAK/ERK/NF-κB signaling significantly suppressed the cell proliferation or migration of high stiffness cultured liver cancer cells, suggesting the crucial role of FAK/ERK/NF-κB signals in high stiffness induced liver cancer development. However, limited anticancer effects of those inhibitors was observed in low stiffness cultured SMMC-7721 cells, which might be caused the slight expression of FAK/ERK/NF-κB in tumor cells. Also, the potential cytotoxicity caused by inhibitors might be addressed. Overall, our results further confirmed the underlying mechanism of high matrix stiffness induced liver cancer development, and pointed out novel targets for liver cancer diagnose.

Owing to the crucial role of integrin in liver cancer, we combined integrin inhibitor GLPG0187 with chemotherapeutic ADM/DDP for improved outcome in liver cancer treatment. Current findings have suggested that small integrin molecule inhibitors or integrin neutralizing antibodies could efficiently suppress tumor growth and disrupt angiogenesis in mice models. Meanwhile, blockade of integrin signals could also suppress distant metastasis or peripheral tissue invasion in melanoma due to inhibition of epithelial-mesenchymal transition process in tumor cells [43]. More importantly, several integrin inhibitors, such as integrin αvβ3 inhibitor cilengitide, are reported to suppress tumor progression in clinical trials and efficiently improved the outcome in patients [44]. Here, our study further determined the anticancer effects of integrin inhibitor GLPG0187, which significantly hindered liver cancer development and prolonged survival time of tumor bearing mice. Consistently, the detection of integrin β1 or matrix stiffness might serve as potential indicators in clinical liver cancer progression analysis and diagnosis.

## Conclusions

Our work described the curial role of matrix stiffness in liver cancer, which was dependent on the activation of integrin β1/FAK/ERK1/2/ NF-κB signaling pathway. Suppression of integrin signals efficiently strengthened anticancer effects of chemotherapy, which described novel insight in adjuvant therapy of liver cancer.

## Supplementary Information

**Additional file 1.**

## Data Availability

The sequencing data generated in this study was deposited in figshare (DOI: 10.6084/m9.figshare.16621849. Private link: https://figshare.com/s/1536ac8ae85fa4aefa69). The anonymized data used and/or analyzed during the current study are available from the corresponding author on reasonable request.

## Abbreviations

HCC: Human hepatocarcinoma cell
ATCC: American Type Culture Collection
ADM: Adriamycin
DDP: cis-diamine dichloro platinum
PA: Polyacrylamide
AFM: Atomic force microscopy
PMSF: Phenylmethylsulfonyl fluoride
LD: Low degree
HD: High degree
LS: Low stiffness
HS: High stiffness
