# Supplementary material for: Inhibition of matrix stiffness relating integrin β1 signaling pathway inhibits tumor growth in vitro and in hepatocellular cancer xenografts
Source: BMC Cancer. 2021 Nov 25;21:1276. doi: 10.1186/s12885-021-08982-3 (PMC8620230; doi:10.1186/s12885-021-08982-3)
Supplement: Supplementary file 1 — Additional file 1. [file 12885_2021_8982_MOESM1_ESM.zip › full blot.docx]

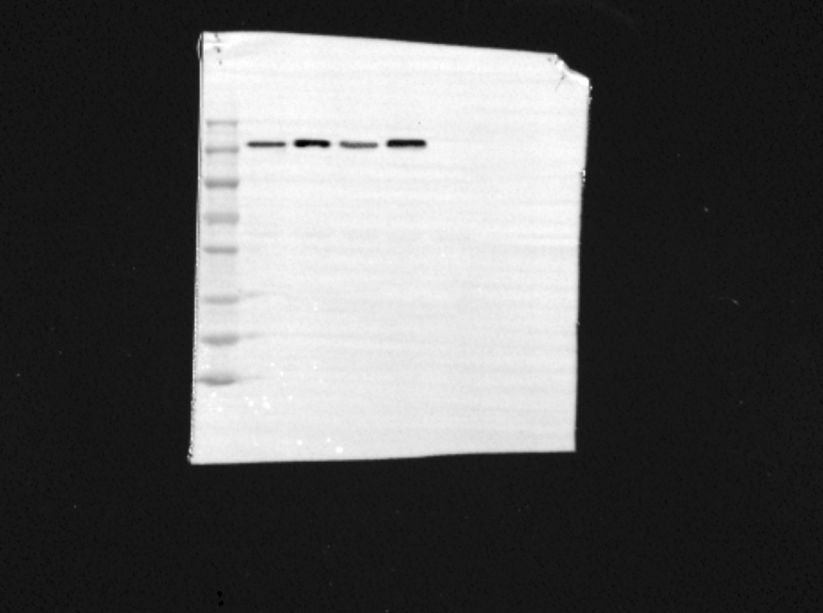


Fig2b itgb1


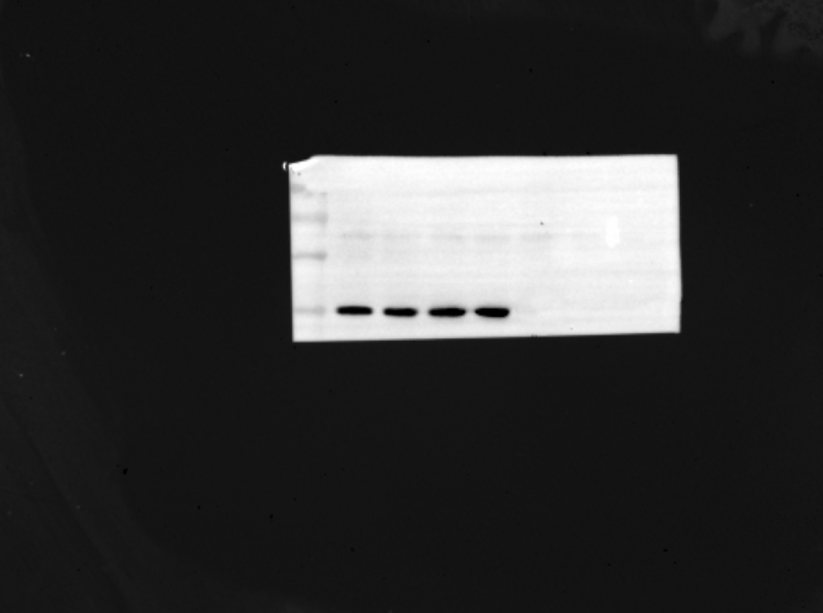


Fig2b actin


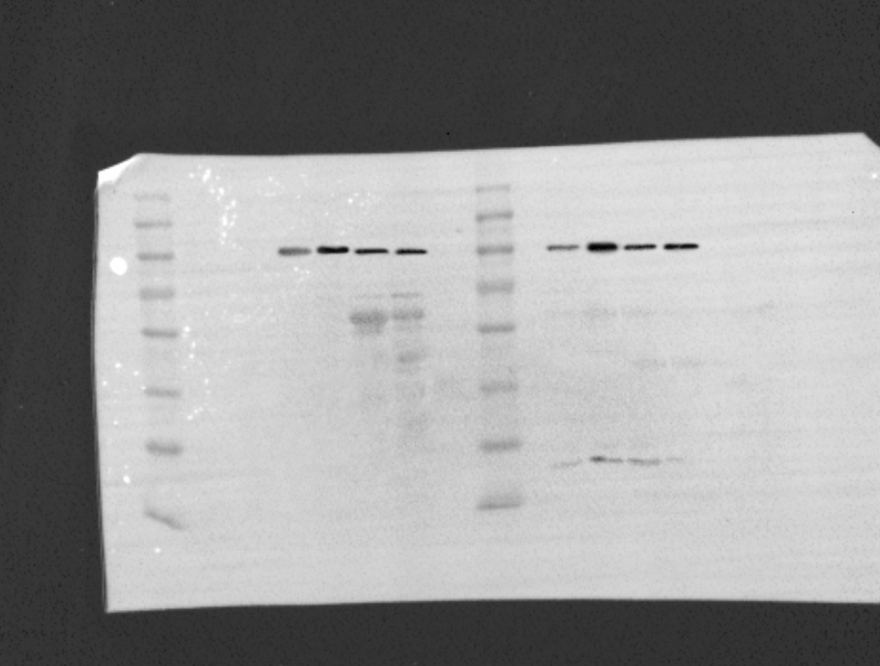


Fig 3a p-FAK (left)

Fig S1a p-FAK (right)


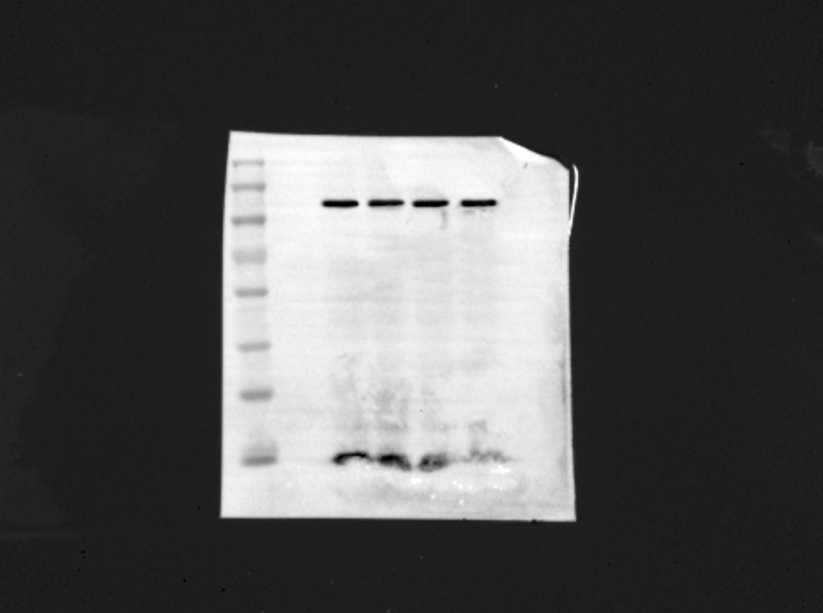


Fig 3a total-FAK


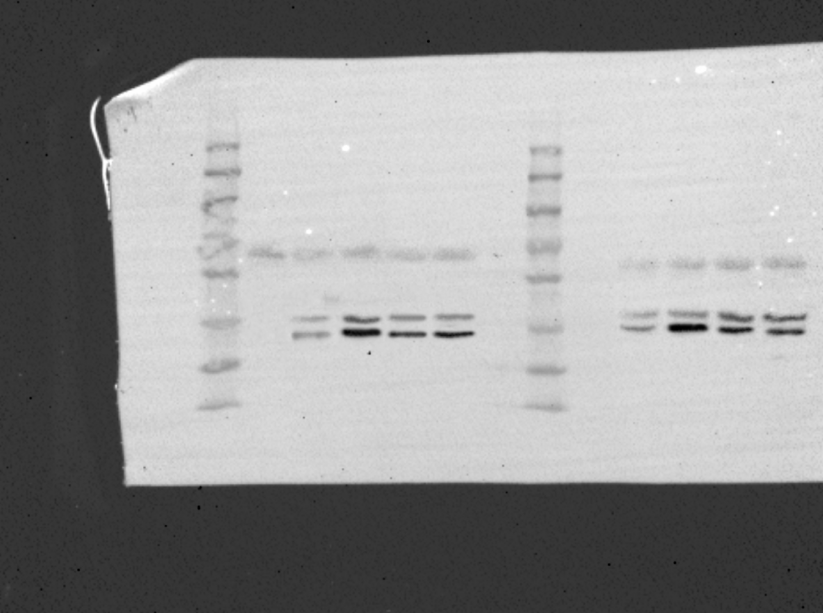


Fig 3a p-ERK (right)

Fig S1a p-ERK (left)


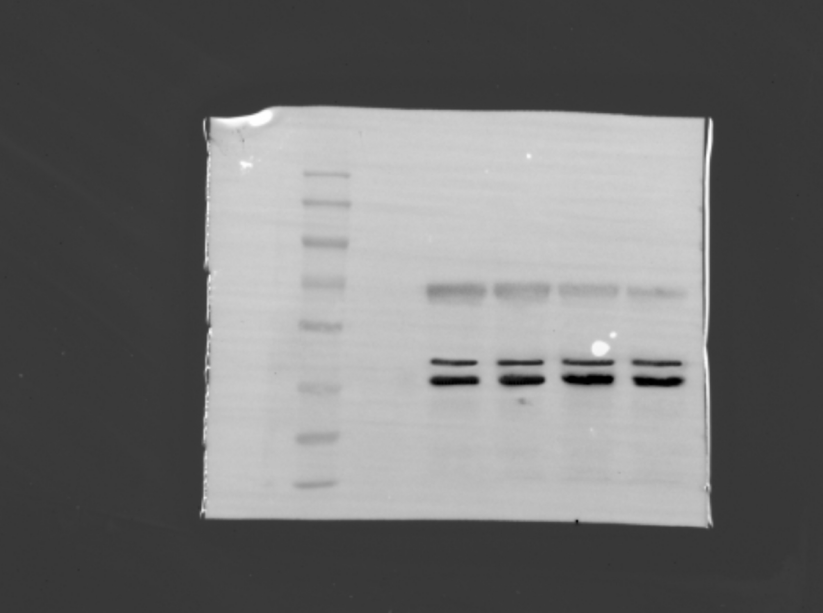


Fig 3a total-ERK


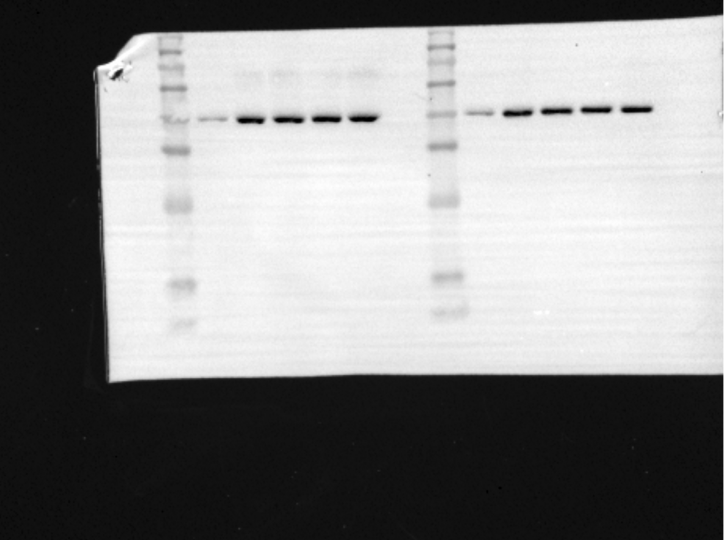


Fig 3a actin (left)

Fig S1a actin (right)


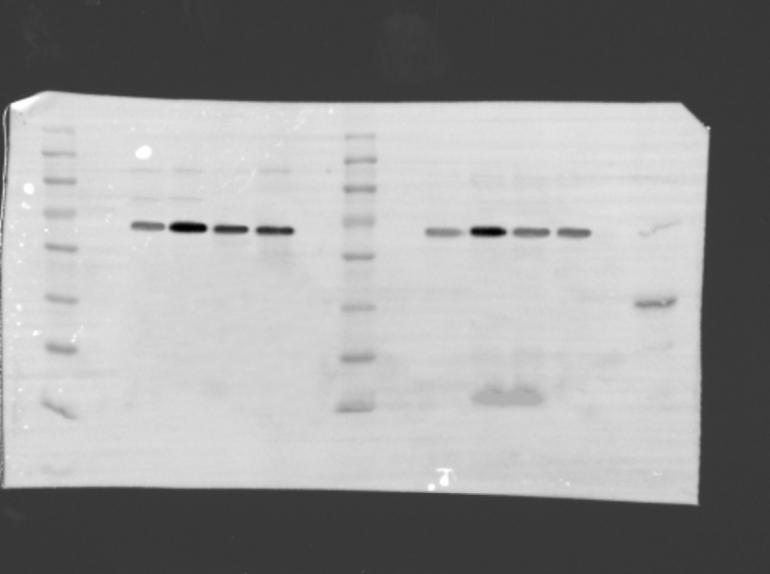


Fig 3e nf-kb (right)

Fig S1e nf-kb (left)


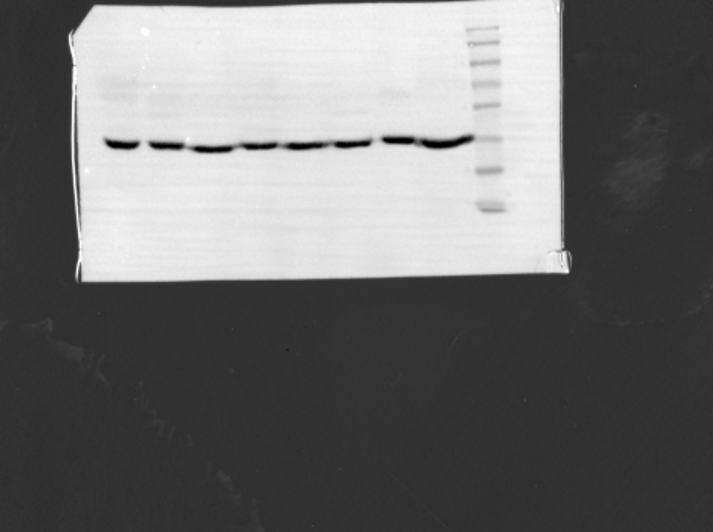


Fig 3e actin (left)

Fig S1e actin (right)


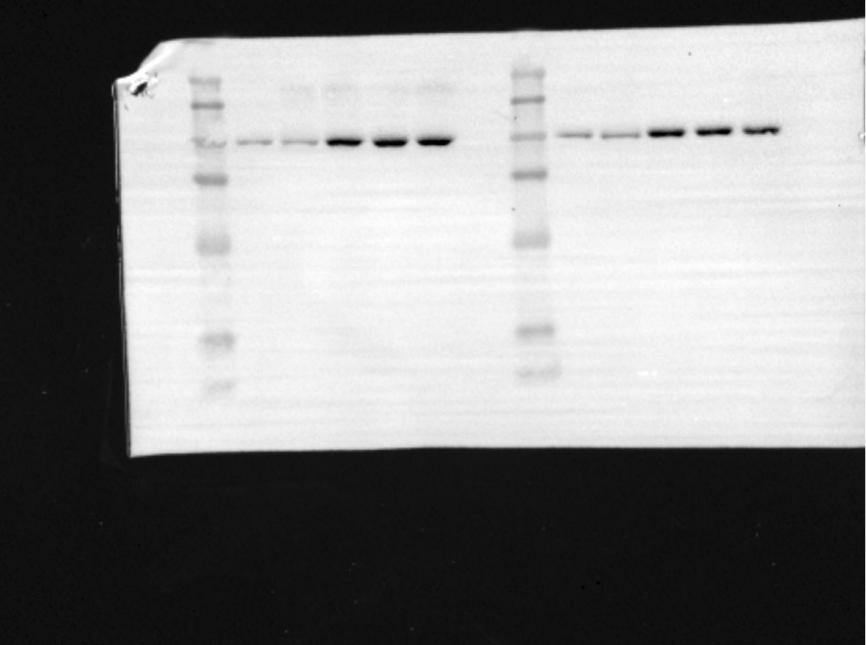


Response-itgb1


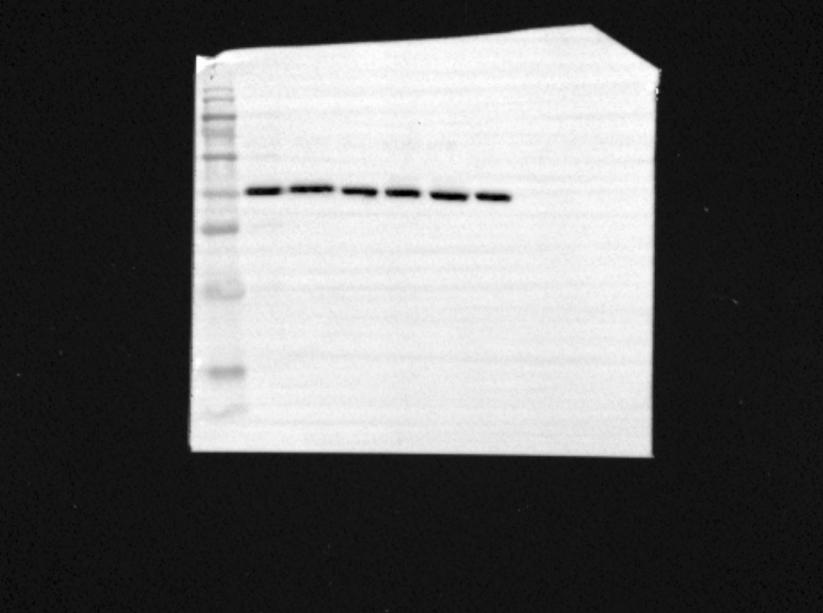


Response-actin
