# Supplementary material for: Inhibition of matrix stiffness relating integrin β1 signaling pathway inhibits tumor growth in vitro and in hepatocellular cancer xenografts
Source: BMC Cancer. 2021 Nov 25;21:1276. doi: 10.1186/s12885-021-08982-3 (PMC8620230; doi:10.1186/s12885-021-08982-3)
Supplement: Supplementary file 1 — Additional file 1. [file 12885_2021_8982_MOESM1_ESM.zip › supplementary file.docx]

**Supplementary Information**

**
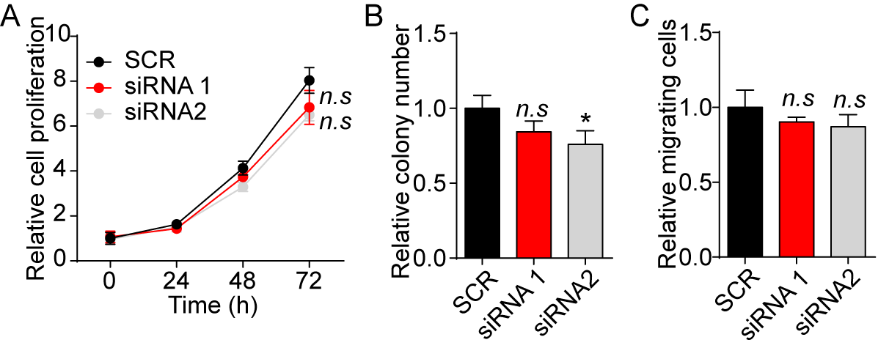
**

**Supplementary Figure 1** A, the relative cells proliferation of SMMC-7721 cells with integrin β1 silenced by siRNA1, siRNA2 or not, cultured in in tunable PA hydrogels with a stiffness of 12 kPa. E, the relative colony number of SMMC-7721 cells pre-cultured in in tunable PA hydrogels with a stiffness of 12 kPa, with integrin β1 silenced by siRNA1, siRNA2 or not. F, the relative migrative cells number of SMMC-7721 cells pre-cultured in in tunable PA hydrogels with a stiffness of 12 kPa, with integrin β1 silenced by siRNA1, siRNA2 or not. Data represent mean ± SD, *P<0.05, n.s no significant difference.

**Supplementary Figure 2**


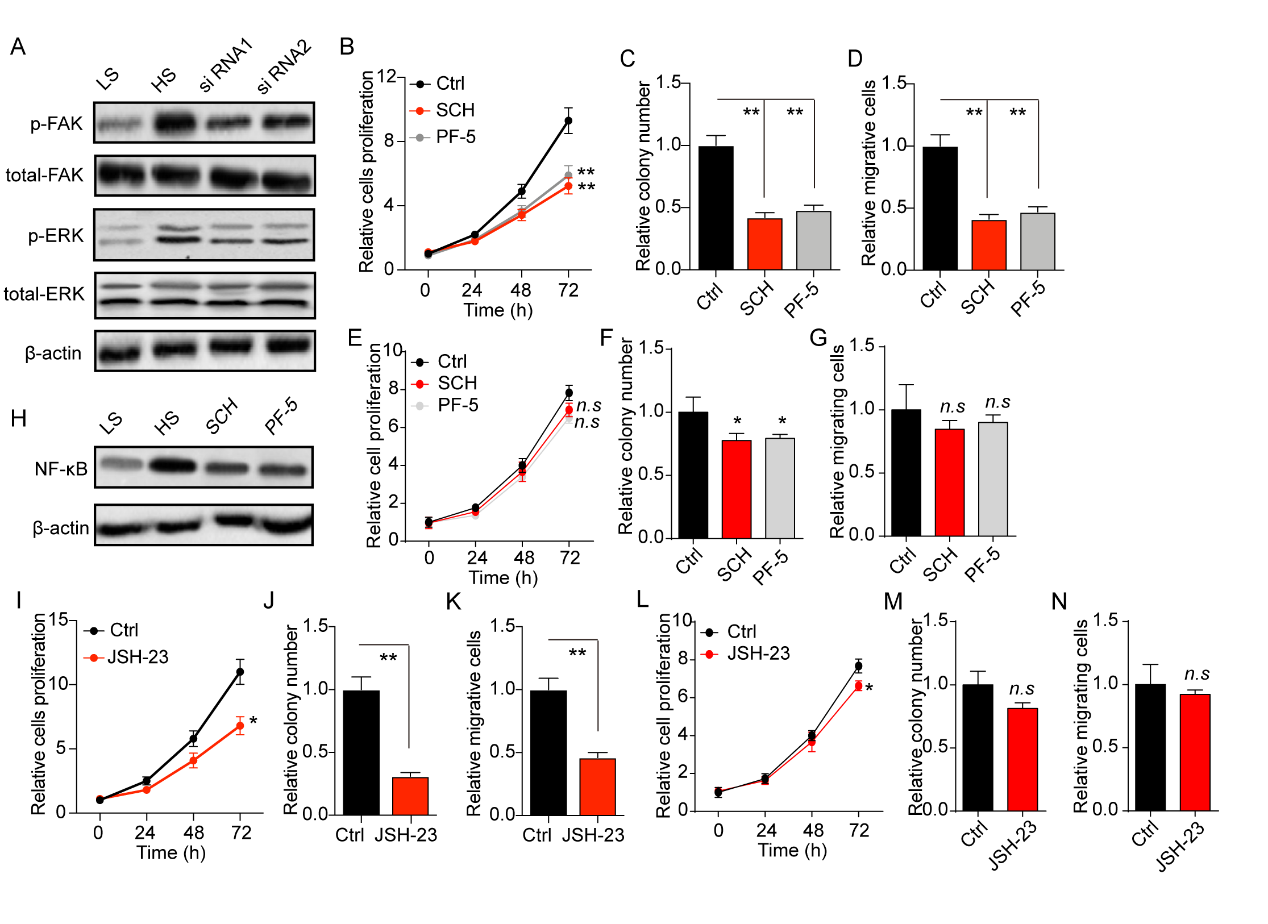


A, the protein expression level of p-FAK, total FAK, p-ERK1/2, total ERK in HepG2 cells cultured in tunable PA hydrogels with low stiffness (12 kPa) and high stiffness (16 kPa), as well as the integrin β1 silenced HepG2 cells cultured in high stiffness, measured by western blot. B, C, D, the relative cells proliferation (B), the relative colony number (C), the relative migrating cell number (D) of HepG2 cells treated with PBS, SCH772984 (2 nM) or PF-573228 (3 nM) respectively as indicated, cultured in high matrix stiffness. E, F, G, the relative cells proliferation (E), the relative colony number (F), the relative migrating cell number (G) of SMMC-7721 cells treated with PBS, SCH772984 (2 nM) or PF-573228 (3 nM) respectively as indicated, cultured in low matrix stiffness. H, the protein expression level of NF-κB in HepG2 cells cultured in tunable PA hydrogels with low stiffness (12 kPa) and high stiffness (16 kPa), as well as the integrin β1 silenced HepG2 cells cultured in high stiffness, measured by western blot. I, J, K, the relative cells proliferation (I), the relative colony number (J), the relative migrating cell number (K) of HepG2 cells treated with PBS, JSH-23 (5 μM) respectively, cultured in high matrix stiffness. L, M, N, the relative cells proliferation (L), the relative colony number (M), the relative migrating cell number (N) of SMMC-7721 cells treated with PBS, JSH-23 (5 μM) respectively, cultured in low matrix stiffness. SCH, SCH772984. PF-5, PF-573228. Data represent mean ± SD, *P<0.05, **P<0.01, n.s no significant difference.
